# The lectin-like protein 1 in *Lactobacillus rhamnosus* GR-1 mediates tissue-specific adherence to vaginal epithelium and inhibits urogenital pathogens

**DOI:** 10.1038/srep37437

**Published:** 2016-11-21

**Authors:** Mariya I. Petrova, Elke Lievens, Tine L. A. Verhoeven, Jean M. Macklaim, Gregory Gloor, Dominique Schols, Jos Vanderleyden, Gregor Reid, Sarah Lebeer

**Affiliations:** 1 KU Leuven, Centre of Microbial and Plant Genetics, Leuven, Belgium; 2 University of Antwerp, Department of Bioscience Engineering, Antwerp, Belgium; 3 The Lawson Health Research Institute London, Canada Research and Development Centre for Probiotics, London, ON, Canada; 4 University of Western Ontario, London, ON, Canada; 5 KU Leuven, Rega Institute for Medical Research, Leuven, Belgium

## Abstract

The probiotic *Lactobacillus rhamnosus* GR-1 has been documented to survive implantation onto the vaginal epithelium and interfere with urogenital pathogens. However, the molecular mechanisms involved are largely unknown. Here, we report for the first time the construction of dedicated knock-out mutants in *L. rhamnosus* GR-1 to enable the study of gene functions. In a search for genes responsible for the adherence capacity of *L. rhamnosus* GR-1, a genomic region encoding a protein with homology to lectin-like proteins was identified. Phenotypic analyses of the knock-out mutant of *L. rhamnosus* GR-1 revealed a two-fold decreased adhesion to the vaginal and ectocervical epithelial cell lines compared to wild-type. In contrast, the adhesion to gastro-intestinal epithelial (Caco2) and endocervical cell lines (Hela and End1/E6E7) was not drastically affected by the mutation, suggesting that the LGR-1_Llp1 lectins mediates tissue tropism. The purified LGR-1_Llp1 protein also inhibited biofilm formation and adhesion of uropathogenic *Escherichia coli*. For the first time, an important role for a novel lectin-like protein in the adhesion capacity and host cell-specific interaction of a vaginal probiotic *Lactobacillus* strain has been discovered, with an additional role in pathogen inhibition.

Probiotics are defined as “live microorganisms which, when administrated in adequate amounts, confer a health benefit on the host”^1^. *Lactobacillus rhamnosus* GR-1 is a well-known probiotic strain isolated from a healthy female urethra. This strain has been shown to adhere well to urogenital epithelial and vaginal cells *in vitro*^2^, and to temporarily colonize the human vagina^3^ and intestine following oral uptake^4^,^5^. The fact that oral application of *L. rhamnosus* GR-1 can result in vaginal colonization^3^,^4^,^5^ is of interest in view of the natural ascension of lactobacilli from the gastro-intestinal tract to the vagina. The ability of *L. rhamnosus* GR-1 to inhibit the growth and adhesion of urogenital pathogens is believed to be important in its probiotic activity. This activity is well documented *in vitro* for pathogens such as *Escherichia coli*^6^,^7^, *Enterococcus*^8^, *Gardnerella vaginalis*^9^, *Atopobium vaginae*^9^ and *Candida albicans*^10^. However, the molecular mechanisms by which this vaginal probiotic strain interacts with pathogenic bacteria and host cells are largely unknown, despite this strain being a model for vaginal probiotics.

Studies of gastro-intestinal probiotic lactobacilli indicate key roles for surface molecules in host interactions^11^. An interesting class of surface molecules are the lectins, i.e. proteins that bind carbohydrates with high specificity, but do not modify them. Lectins are well characterized in animals and plants^12^,^13^, while the information in bacteria is relatively poor. The best documented bacterial lectins are present in uropathogenic bacteria, such as the FimH adhesin located at the tip of type 1 pili of the uropathogenic *E. coli* (UPEC) where they play a role in attachment to urothelium by binding to mannosylated glycoreceptors^14^.

In the present study, we aimed to better understand the molecular factors that contribute to *L. rhamnosus* GR-1 vaginal adherence, immunomodulation and pathogen inhibition. Because of the presence of various glycans on the vaginal mucosa and surfaces of pathogenic microorganisms, we investigated whether lectin-like proteins could play a role in adhesion and immunomodulation capacity of *L. rhamnosus* GR-1, and in its capacity to prevent uropathogenic *E. coli* infections.

## Results

### Identification and annotation of the *LGR1*_*llp1* gene encoding the lectin-like protein 1

**Figure 1:**
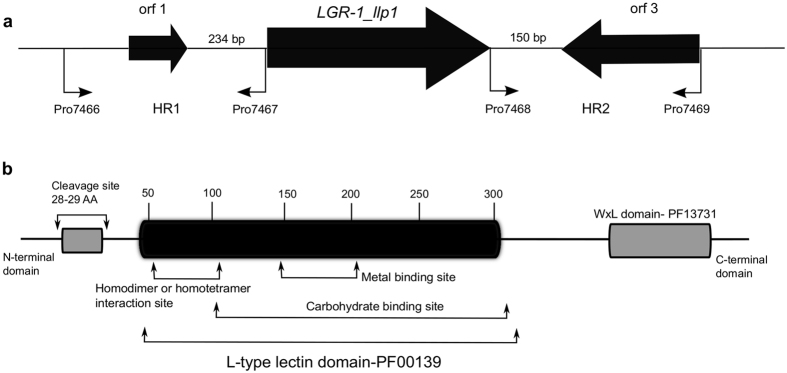
Genomic region and protein domain organization of LGR1_Llp1. **(a**) The genomic region of the *LGR1_llp1* gene with the surrounding genes. The first ORF is predicted to encode a putative binding protein and the third divergent ORF encodes a putative transmembrane transporter. The primer binding sites for amplifying the HR1 and HR2 are indicated with arrows. (**b)** Putative protein domain structure of LGR1_Llp1. The lectin like domain (PF00139, clan CL0004) constitutes ca. 250 amino acids and is predicted to contain three specific sites (1) one responsible for specific carbohydrate recognition; (2) a metal-binding site and (3) the homodimer or homotetramer interaction sites. The cleavage site in the *N*-terminal domain required for removal of the signal leader peptide and export of the protein out of the cells is also shown. The *C*-terminal domain WxL domain (PF13731) putatively responsible for the anchoring the protein on the cell wall^53^,^54^ is also depicted. Genes and protein structure are not represented at scale.

To identify genes encoding putative lectin-like proteins, the draft genome sequence of *L. rhamnosus* GR-1 was screened for the presence of open reading frames (ORFs) containing a lectin L-type domain (PF00139). A 4060 bp genomic region was identified (Fig. 1a) containing a 2040 bp sequence encoding a polypeptide of 680 amino acid residues, with an *N*-terminal Legume-type (L-type) lectin domain (PF00139) and a *C*-terminal WxL domain (PF13731) (Fig. 1b). We annotated this gene sequence as *LGR1_llp1,* encoding the putative lectin-like protein 1. The L-type lectin domain represents approximately 250 amino acid residues in length and is found in several cell surface proteins of Gram-positive bacteria^15^. The C-terminal WxL domain, detected also in proteins from several other Gram-positive bacteria, is suggested to be responsible for the non-covalently anchoring of proteins to the microbial surface, possibly by interaction with the peptidoglycan^16^.

### LGR1_Llp1 mediates tissue-specific adhesion of *L. rhamnosus* GR-1 to vaginal epithelial cells

In order to develop a DNA transformation protocol for *L. rhamnosus* GR-1, two different plasmids were used: the *Lactobacillus* genome integrating pEM40 vector^17^ and the self-replicative *Lactobacillus* pLAB1301 vector^18^. The electroporation protocol for *L. rhamnosus* GG^19^ was used as a starting point. Both plasmids could be transformed with a similar efficiency as for *L. rhamnosus* GG, with an electroporation efficiency of ca. ~1, 7 × 10^6^ CFU/μg DNA for pLAB1301 and ~1 × 10^5^ CFU/μg DNA for pEM40. This is in agreement with the latter being an integrative plasmid in *L. casei* and *L. rhamnosus* strains^17^, thus requiring an integration step in the genome in addition to efficient transformation, resulting in lower efficiency.

**Figure 2:**
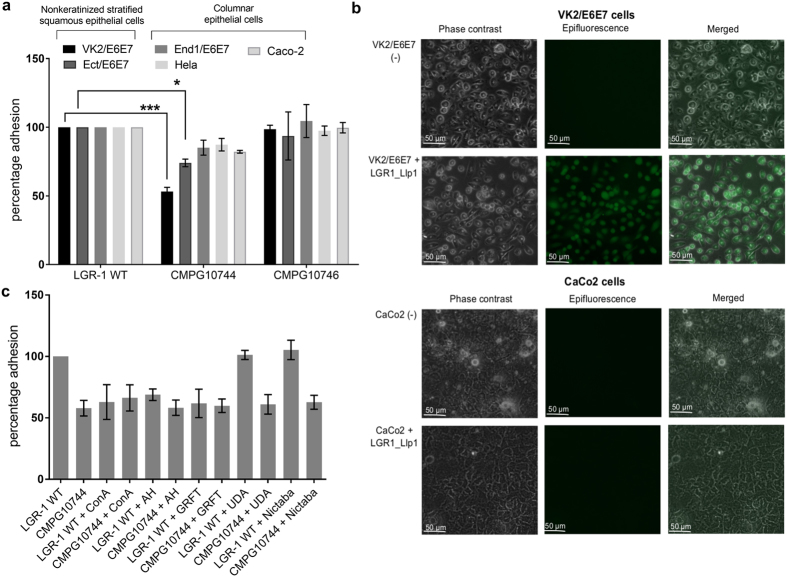
Role of LGR1_Llp1 in the adhesion capacity of *L. rhamnosus* GR-1 to epithelial cells. **(a)** Adhesion to different epithelial cell lines. Five different types of epithelial cell lines (VK2/E6E7, Ect/E6E7, End1/E6E7, Hela, Caco-2) were used to determine epithelial specific binding of *L. rhamnosus* GR-1 and the corresponding *LGR1_llp1* (CMPG10744) mutant. The error bars represent standard deviations of three independent experiments. The dataset comparisons (mutant pairwise to wild type) are considered significant (p < 0.05 indicated with one asterisks in the figure and p < 0.001indicated with three asterisks in the figure). (**b)** Binding of the FITC-labelled lectin domain of LGR1_Llp1 to VK2/E6E7 and Caco-2 cells. (**c)** Adhesion to VK2/E6E7 cells after treatment with ConA, AH, GRFT, UDA and Nictaba. The results are expressed as the percentage of the bacterial cells adhering to VK2/E6E7 cells after addition of the lectins versus the condition without addition of lectins. The error bars represent standard deviations of three independent experiments.

By using the optimized electroporation protocol, a knock-out *LGR1_llp1* mutant was constructed by double homologous recombination. The correct allelic replacement event in *LGR1_llp1*mutant CMPG10744 with tetracycline resistant antibiotic cassette was confirm by PCR and Southern hybridization. Subsequently we investigate the role of Llp1 protein in the adhesion capacity of *L. rhamnosus* GR-1. The *LGR1_llp1* mutant CMPG10744 showed a significant (p = 0.0006) ca. two-fold reduction in adhesion capacity to the vaginal epithelial cell line VK2/E6E7 compared to *L. rhamnosus* GR-1 wild type (Fig. 2a). In addition, the *LGR1_llp1* mutant CMPG10744 showed also a significant (p = 0.04) reduction in adhesion capacity with 26% to the ectocervical epithelial cells Ect/E6E7, which is also nonkeratinized stratified squamous epithelium (Fig. 2a). To confirm the genotype-phenotype relation for the *LGR1*_*llp1* gene, mutant CMPG10744 was subsequently complemented by re-introducing the *LGR1_llp1* gene. This complemented strain CMPG10746 showed complete restoration of the adhesion phenotype, reaching the same adhesion capacity levels as the wild type (Fig. 2a). We subsequently investigated whether the LGR1_Llp1 protein is also involved in the adhesion capacity to other, simple columnar epithelial cells lines such as the endocervical End1/E6E7, the model cervical cancer Hela cell line and the colon carcinoma Caco-2 cell line. Interestingly, the adhesion capacity of *LGR1_llp1* CMPG10744 was only slightly but not significantly reduced for the intestinal Caco2 cells line, the endocervical End1/E6E7 cell line and the Hela cell line as compared to the *L. rhamnosus* GR-1 wild type (Fig. 2a). This suggests that LGR1_Llp1 mediates tissue tropism with specificity towards vaginal epithelial and stratified squamous epithelial cells.

To confirm tissue specific adhesion by LGR1_Llp1, the lectin domain of LGR1_Llp1 was expressed in *E. coli* and purified. Subsequently, the binding of the FITC- labelled lectin domain of LGR1_Llp1 was explored by fluorescence microscopy for VK2/E6E7 and Caco-2 cells grown on cover slips. In contrast to Caco-2 cells, the lectin domain recognized and bound strongly to the VK2/E6E7 epithelial cells (Fig. 2b), suggesting indeed that the lectin plays a role in the well-documented adhesion capacity of *L. rhamnosus* GR-1.

### Indication for a lectin-like role for Llp1 in adhesion of GR-1 to mannosylated vaginal epithelial cells

To validate that the adhesion role of LGR1_Llp1 is due to its sugar-binding capacity, we performed several complementary experiments. First, we designed a competition experiment with lectins with known specificity. After preincubation of VK2/E6E7 cells with the α (1,6)- α (1,3) mannose- specific lectin ConA, α(1,2) mannose- specific AH and GRFT, but not with (GlcNAc)n specific UDA and Nictaba, a significant ca. two-fold reduction in the adhesion capacity of *L. rhamnosus* GR-1 wild type was observed (Fig. 2c). On the other hand, the adhesion capacity of the *LGR1_llp1* mutant CMPG10744 did not vary and was always ca. 40% of wild-type. These results indicate that the ConA, AH and GRFT block important mannosylated receptors on the cell surface of VK2/E6E7 and that LGR1_Llp1 possibly competes with these lectins for the same receptors on these vaginal epithelial cells.

**Figure 3:**
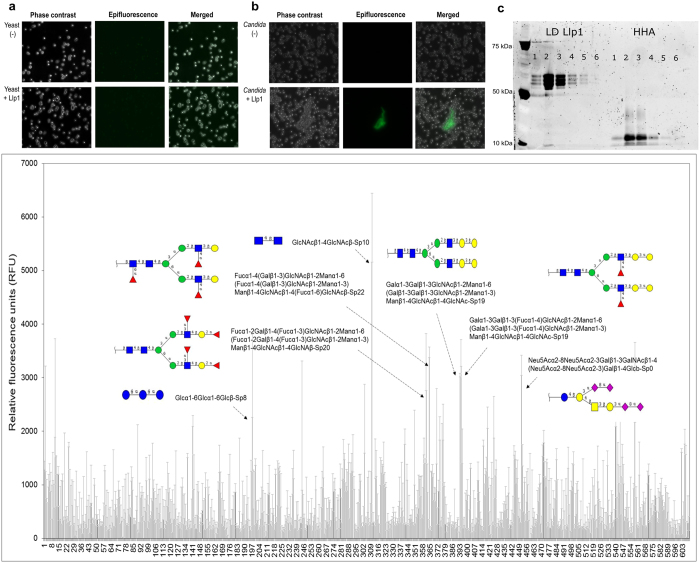
Carbohydrate specificity of the lectin domain of LGR1_Llp1. **(a,b)** Fluorescent images of the agglutination assay of *S. cerevisiae* BY4741 and *C. albicans* SC5314 in the presence of the FITC labelled lectin domain of LGR1_Llp1. (**c)** Proteins that bound to sugar-coated Sepharose beads were separated by SDS-PAGE. Sepharose beads were coated with mannan (lane 2), D-mannose (lane 3), D-glucose (lane 4), D-fucose (lanes 5) GluNAc (lane 6) or not coated with any sugar (lane 1, used as negative control). (**d)** Mammalian glycan array used to determine the carbohydrate binding specificity of the lectin domain of Llp1. Glycans to which the FITC labelled lectin domains show the strongest binding are depicted.

Subsequently, agglutination assays with mannan-containing yeasts were performed to confirm the mannose specificity of LGR1_Llp1. The FITC-labelled LGR1_Llp1 did not aggregate the yeast *Saccharomyces cerevisiae* BY4741 (Fig. 3a), but did lead to aggregation of the important opportunistic pathogen *Candida albicans* SC5314 (Fig. 3b) as compared to the negative control. The affinity of the purified lectin domains towards various sugars was subsequently further examined by pull-down sugar-binding assays with beads coated with purified mannan, D-mannose, D-glucose, D-fucose and N-acetylglucoseamine (GlcNAc) (Fig. 3c). *Hippeastrum* hybrid lectin (HHA), a well-known mannose-specific plant lectin was used as a positive control. The purified lectin domain of Llp1 showed the highest binding to mannan and mannose (Fig. 3c, lane 2 and lane 3 respectively). There was no significant binding to any of the other sugars tested (glucose, fucose and GlcNAc).

The purified FITC-labelled lectin domain was then used for glycan array screening against a panel of 609 mammalian N-glycan structures. These data indicate that the lectin domain of Llp1 recognizes some complex N-glycans, such as: (i) GlcNAcβ1-4GlcNAcβ-Sp10 (ii) Fucα1-4(Galβ1-3)GlcNAcβ1-2Manα1-6(Fucα1-4(Galβ1-3)GlcNAcβ1-2Manα1-3)Manβ1-4GlcNAcβ1-4(Fucα1-6)GlcNAcβ-Sp22; (iii) Galα1-3Galβ1-3(Fucα1-4)GlcNAcβ1-2Manα1-6(Galα1-3Galβ1-3(Fucα1-4)GlcNAcβ1-2Manα1-3)Manβ1-4GlcNAcβ1-4GlcNAc-Sp19; (iv) Fucα1-2Galβ1-4(Fucα1-3)GlcNAcβ1-2Manα1-6(Fucα1-2Galβ1-4(Fucα1-3)GlcNAcβ1-2Manα1-3)Manβ1-4GlcNAcβ1-4GlcNAβ-Sp20; (v) Neu5Acα2-8Neu5Acα2-3Galβ1-3GalNAcβ1-4(Neu5Acα2-8Neu5Acα2-3)Galβ1-4Glcβ-Sp0 (Fig. 3d).

### LGR1_Llp1 only moderately induces immune responses in vaginal epithelial cells

**Figure 4:**
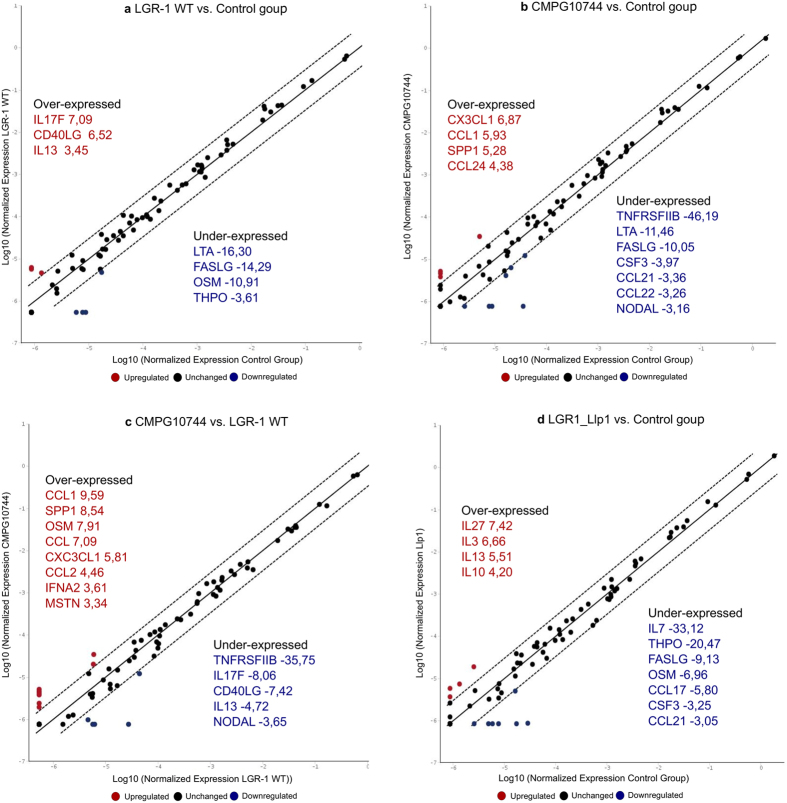
Cytokine and chemokine induction by *L. rhamnosus* GR-1, LGR1_llp1 (CMPG10744) mutant and L-type lectin domain of LGR1_Llp1. The results represent the upregulation (in red), downregulation (in blue) or the unchanged (in black) mRNA expression of 84 cytokines. All the genes upregulated and downregulated with more than 3-fold difference as compared to the negative control VK2/E6E7 cells (**a**,**b** and **d**) or to *L. rhamnosus* GR-1 wild type (**c**) are depicted on the graph.

The host-cell signalling interaction capacity of LGR1_Llp1 in vaginal VK2/E6E7 cells was then investigated by cytokine arrays that explore the upregulation and downregulation of the mRNA expression of selected genes. Interestingly, the mRNA expression level of the majority of cytokines and chemokines tested was unchanged after incubation with the bacterial strains or with the purified lectin domain of LGR1_Llp1 (Fig. 4). However, the lectin domain of LGR1_Llp1 appeared to induce mRNA levels of the anti-inflammatory cytokines IL13 and IL10 and the pro-inflammatory IL27 by more than four-fold. Of note, IL13 mRNA expression was also upregulated by the wild type *L. rhamnosus* GR-1 (Fig. 4a), while it was downregulated by LGR1_Llp1 mutant (Fig. 4c). Both *L. rhamnosus* GR-1 and LGR1_Llp1 also induced a downregulation of the growth factors OSM, FASLG and THPO, as well as of FASLG part of the TNF receptor superfamily. In addition, LGR1_Llp1 induced a strong ca. 33 fold downregulation of IL7 mRNA.

### LGR1_Llp1 inhibits adhesion and biofilm formation of the key urogenital pathogen *E. coli* UTI89

**Figure 5:**
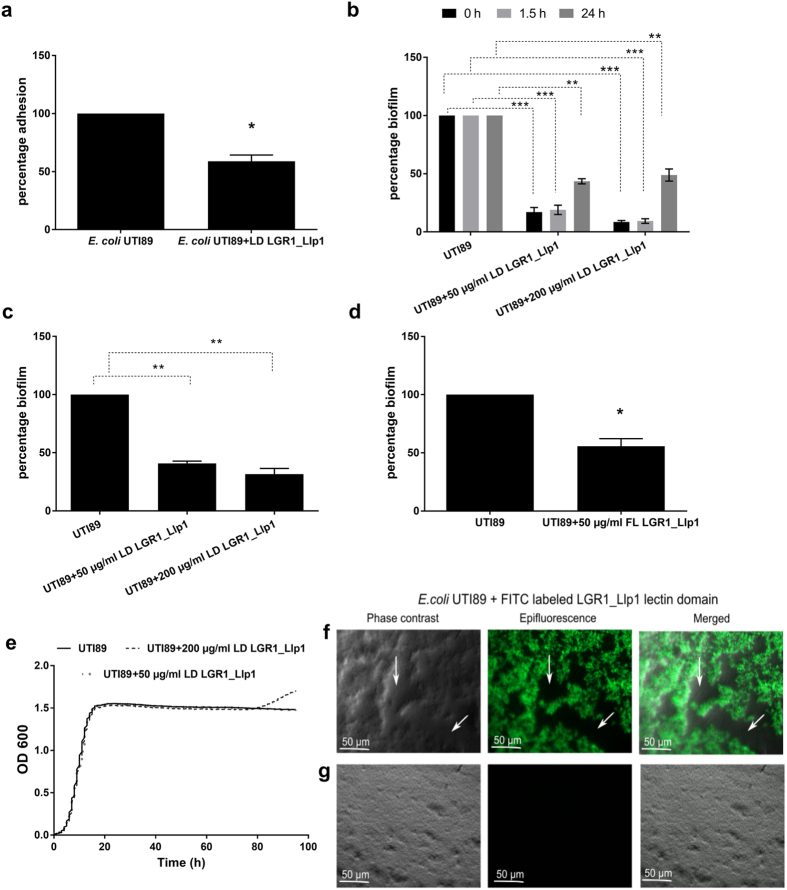
Role of LGR1_Llp1 in inhibiting adhesion and biofilm formation of *E. coli* UTI89. (**a)** Effect of the lectin domain (LD) of LGR1_Llp1 on the adhesion of *E. coli* UTI89 after pre-incubating the bacterial cells with the lectin. (**b)** Effect of the lectin domain of *L. rhamnosus* GR-1 on *E. coli* UTI89 biofilms. The purified lectin domain (LD) of LGR1_Llp1 was added after 0, 1.5 and 24 hours to the biofilms. (**c)** Biofilm formation of *E. coli* UTI89 based on absolute cell counts. Biofilms were grown in 1/20 TSB medium without (control) or with 50 μg/ml and 200 μg/ml of LD of LGR1_Llp1. (**d)** Effect of full length (FL) lectin (50 μg/ml) on *E. coli* UTI89 biofilms added at zero-time point to the biofilms. (**e)** Growth of *E. coli* UTI89 in the presence of lectin domain of LGR1_Llp1 added at concentrations of 50 μg/ml and 200 μg/ml. The error bars represent standard deviations of three independent experiments. The dataset comparisons are considered significant (p < 0.05 indicated with one asterisk in the picture, p < 0.01 indicated with two asterisks or p < 0.001 indicated with three asterisks). (**f)** Biofilms of *E. coli* UTI89 grown with 50 μg/ml of FITC-labeled lectin domain of LGR1-Llp1 and (**g)** alone without adding lectin in 1/20 TSB medium (negative control). Holes in biofilms are indicated with arrows.

Since pathogen inhibition is a key hallmark of probiotic bacteria, whether LGR1_Llp1 could block adhesion and biofilm formation of UPEC was investigated. When *E. coli* UTI89 was pre-incubated with the lectin domain of LGR1_Llp1 at concentration of 50 μg/ml, it caused a significant almost two-fold reduction in the adhesion capacity of *E. coli* UTI89 to VK2/E6E7 cells (Fig. 5a), indicating a role in pathogen exclusion from host cells.

When the purified lectin domain of LGR1_Llp1 was added at the beginning of biofilm development at concentration 50 and 200 μg/ml, a reduction to 17% and 8% of the control situation was observed respectively (Fig. 5b). When the lectin domain of LGR1_Llp1 at 50 μg/ml was added after 1.5 or 24 h, a reduction to 18% and 43% respectively in the biofilm formation was still observed compared to the control (Fig. 5b). The absolute CFU counts of *E. coli* UTI89 in the formed biofilms were in agreement. The lectin domain of LGR1_Llp1 caused then a significant reduction of *E. coli* UTI89 biofilm to ca. 40% (for 50 μg/ml concentration) and 30% (for 200 μg/ml concentration) of the undisturbed pathogenic biofilms (Fig. 5c). Since the lectin domain is part of a full-length protein, we subsequently investigated if the full length LGR1_Llp1 (which is more difficult to express and purify in large amounts) also possesses an anti-biofilm activity. Indeed, when the full-length LGR1_Llp1 was added at the beginning of the biofilm formation at concentration of 50 μg/ml, also a ca. two-fold reduction was observed (Fig. 5d). Given the capacity of the lectin to prevent *E. coli* UTI89 biofilms, bioscreens were performed to investigate whether the lectin also shows an antimicrobial effect on growth in suspension. Interestingly, no inhibitory effect on planktonic growth was observed (Fig. 5e).

To explore how the lectin domain structurally interferes with *E. coli* UTI89 biofilm formation, microscopic analyses were performed. Addition of FITC-labeled lectin domain of LGR1_Llp1 at the onset of the biofilm at concentration 50 μg/ml resulted in the formation of loose biofilms with large holes (Fig. 5f), compared to the control without lectins, which formed dense biofilm (Fig. 5g). The lectin domain of LGR1_Llp1 was also shown to be clearly distributed across the biofilm (Fig. 5e).

### LGR1_Llp1 inhibits adhesion but not biofilm formation of *Staphylococcus aureus*

**Figure 6:**
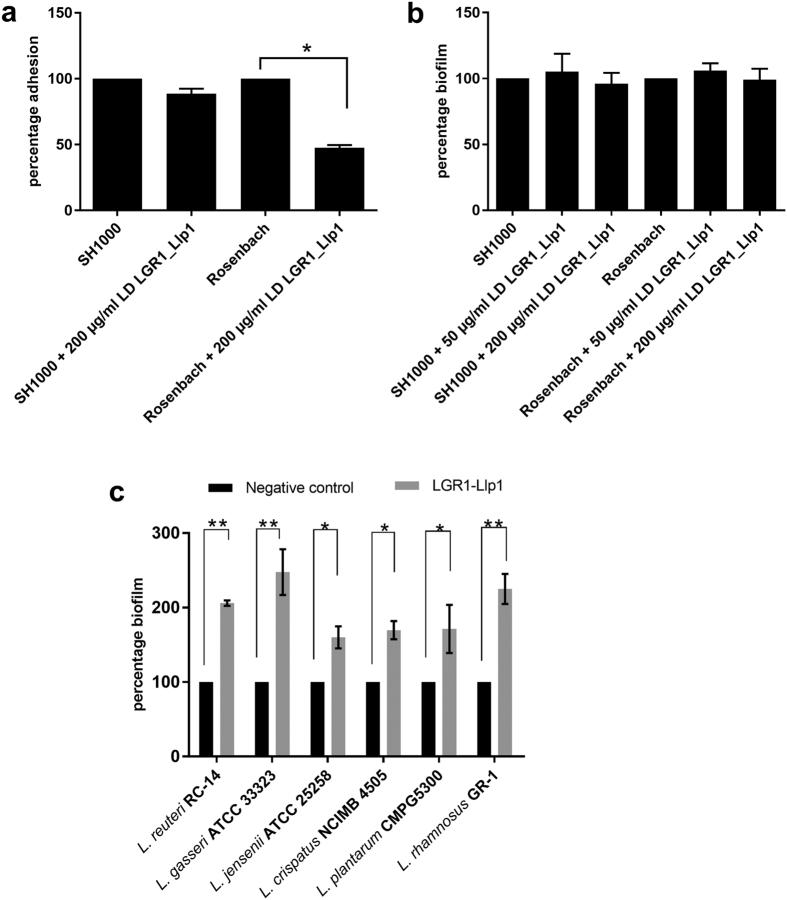
Role of LGR1_Llp1 in inhibiting adhesion and biofilm formation of *S. aureus*. (**a**) effect of lectin domain (LD) of LGR1_Llp1 on the adhesion of *S. aureus* SH1000 and *S. aureus* Rosenbach after pre-incubating the bacterial cells with the lectin domain. (**b**) Effect of the lectin domain of *L. rhamnosus* GR-1 on *S. aureus* SH1000 and *S. aureus* Rosenbach biofilms. The purified lectin domain (LD) of LGR1_Llp1 was added at the zero time point to the biofilms. (**c**) Biofilm formation of vaginal *Lactobacillus* strains. Biofilms were grown in MRS medium without (control) or with 50 μg/ml of LD of LGR1_Llp1. The error bars represent standard deviations of three independent experiments. The dataset comparisons are considered significant (p < 0.05 indicated with one asterisk in the picture, p < 0.01 indicated with two asterisks).

As for *E. coli* UTI89, the effect of the lectin domain of LGR1_Llp1 against the adhesion capacity of *S. aureus* Rosenbach and *S. aureus* SH1000 to VK2/E6E7 cells was investigated by pre-incubating the pathogenic bacterial cells with 50 μg/ml of the lectin domain. A two-fold reduction in the adhesion of *S. aureus* Rosenbach was observed compared to the control treatment (Fig. 6a). No significant differences were notable for LGR1_Llp1 on the biofilm capacity of *S. aureus* Rosenbach and *S. aureus* SH1000 as compared to the negative control (Fig. 6b).

### LGR1_Llp1 increases biofilm formation of vaginal *Lactobacillus* species

Potential new anti-bacterial agents should not affect the beneficial *Lactobacillus*-dominated vaginal microbiota. Of interest, biofilm formation of the vaginal *Lactobacillus* strains tested here was shown to increase in the presence of LGR-1_Llp1. Biofilm formation of *L. reuteri* RC-14 and *L. gasseri* ATCC 33323 increased significantly ca. two-fold and 2.5-fold respectively when LGR1_Llp1 was added in the initial steps of the biofilm formation (Fig. 6c). Furthermore, a significant 1.6 to 1.7-fold increase in biofilm formation was observed for *L. jensenii* ATCC 25258, *L. crispatus* NCIMB 4505 and *L. plantarum* CMPG5300 respectively. Finally, the biofilm formation of *L. rhamnosus* GR-1 itself was also significantly increased with 1.25-fold when LGR1_Llp1 was added (Fig. 6c).

## Discussion

The vaginal ecosystem represents a special niche for the application of probiotic lactobacilli, because of the natural dominance of *Lactobacillus* species in healthy reproductive-age females and the link between *Lactobacillus* disappearance and disease phenotypes^20^,^21^. Therefore, in this study we biochemically and genetically identified and characterized a novel lectin-like protein LGR1_Llp1 from the vaginal probiotic strain *L. rhamnosus* GR-1. The lectin was shown to mediate vaginal niche-related functions for *L. rhamnosus* GR-1, including tissue tropism-mediated adhesion to vaginal and ectocervical epithelial cells and immunomodulation. In addition, the lectin domain showed to have a unique capacity to inhibit adhesion and biofilm formation of vaginally-associated pathogens, without affecting the normal *Lactobacillus*-dominated microbiota.

First, we were able to successfully optimize a protocol for electroporation for the construction of a dedicated knock-out mutant in *L. rhamnosus* GR-1. This report describes the development of genetic tools that permit mechanistic investigations of the probiotic strain *L. rhamnosus* GR-1. Phenotypic analyses of knock-out mutants have the advantage that the function of cell-surface molecules such as adhesions and lectins can be studied *in situ* on the surface of live bacteria in their physiological context. An isogenic *LGR1_llp1* mutant was constructed in a gene encoding a putative lectin-like protein. This *LGR1_llp1* mutant showed a significantly reduced adhesion capacity to vaginal and ectocervical epithelial cells, but not to endocervical, cervical carcinoma cells and colon carcinoma cells. Similar results were also observed when we investigated the binding capacity of the FITC-labelled LGR1_Llp1. This suggests that the LGR1_Llp1 protein provides tissue tropism to *L. rhamnosus* GR-1, likely determined by the presence of different glycosylated structures on the cell membrane of these epithelial cells. The fact that LGR_Llp1 preferentially binds to sugar residues on vaginal epithelial cells, and not intestinal cells, may promote the natural ascension and easier transit from the gastrointestinal tract to the vaginal epithelium by this vaginal strain. Of note, both Hela and Caco-2 cell lines were recently shown to have a similar complex glycosylation profile^22^. The glycan structure of surface molecules of VK2/E6E7 cells is not yet well studied. However, previous studies suggest that glucose, mannose and glucosamine are important sugars in the vaginal niche^23^,^24^.

To substantiate the lectin-like role of LGR1_Llp1 in the adhesion capacity of *L. rhamnosus* GR-1, competition experiments with lectins with well-known specificity for adhesion to vaginal epithelial cells were performed. These experiments suggest that LGR1_Llp1 on the cell surface of *L. rhamnosus* GR-1 is involved in the recognition of similar glycosylated receptors on the surface of VK2/E6E7 cells as the tested α(1,6) Man, α(1,3) Man specific ConA and α(1,2) Man specific AH and GRFT lectins. However, studying the exact sugar specificity of LGR1_Llp1 is difficult in the context of the bacterial cells, because various other cell surface molecules may interfere. Therefore, we also determined the sugar specificity of the purified LGR1_Llp1 lectin domain by using yeast agglutination assay, combined with Sepharose beads binding assays and dedicated mammalian glycan array screenings. All these experiments indicate specific binding of LGR1_Llp1 to complex N-glycan structures, in agreement with recent studies on plant lectins showing that their specificity is complex and cannot be merely described by single sugar monomers^25^. The glycan array especially revealed a strong binding of LGR1_Llp1 to GlcNAcβ1-4GlcNAcβ as well as to galactosylated, fucosylated and mannosylated glycans, in agreement with the capacity of LGR1_Llp1 to agglutinate *C. albicans* and bind mannan. For example, LGR1_Llp1 showed binding to Lewis A and Lewis Y terminals present on two of the complex glycans tested positive in the glycan array (Fucα1-4(Galβ1-3)GlcNAcβ1-2Manα1-6(Fucα1-4(Galβ1-3)GlcNAcβ1-2Manα1-3)Manβ1-4GlcNAcβ1-4(Fucα1-6)GlcNAcβ-Sp22 and Fucα1-2Galβ1-4(Fucα1-3)GlcNAcβ1-2Manα1-6(Fucα1-2Galβ1-4(Fucα1-3)GlcNAcβ1-2Manα1-3)Manβ1-4GlcNAcβ1-4GlcNAβ-Sp20 respectively). Lewis A and Lewis Y have been shown to be expressed on various epithelial cells, such as gastrointestinal epithelium, vaginal epithelium, embryonic tissues and saliva^26^,^27^. In addition, LGR1_Llp1 showed also a capacity to bind to the glycosphingolipid GQ1 receptor (Neu5Acα2-8Neu5Acα2-3Galβ1-3GalNAcβ1-4(Neu5Acα2-8Neu5Acα2-3)Galβ1-4Glcb-Sp0, also present also on various epithelial cells, and being an important receptor for bacterial toxins with lectin activity^27^. However, it is important to highlight that the exact sugar specificity of LGR1_Llp1 remains to be further established. For instance, it would be interesting to screen also bacterial glycans, since in this study we could only screen for mammalian glycans with the glycan array. Importantly, bacterial glycans are generally far more diverse than eukaryotic glycoconjugates, as they can show an enormous diversity in monosaccharide building blocks, configuration, conformation and stereochemistry^28^. Nevertheless, the fact that we could elucidate part of the sugar specificity of this bacterial lectin is worth mentioning, since only for a limited number of bacterial lectins, the sugar specificity has been determined, even rarely by glycan array. One important example of a well-characterized bacterial lectin is the soluble lectin from *P. aeruginosa* LecB (also known as PA-IIL). LecB binds to a large variety of fucosylated oligosaccharides, such as α-Fuc 1-2 Gal and β-Gal 1-4 α(Fuc1-3)GlcNAc, as confirmed with glycan array^29^.

Since LGR1_Llp1 mediated the adhesion capacity of *L. rhamnosus* GR-1, we also investigated its role in modulating cytokine responses in VK2/E6E7 cells. Probiotic strains have been reported to promote health by stimulating the host immune response^30^, but such stimulation of vaginal host cells is poorly documented^21^. We did not observe strong cytokine responses when VK2/E6E7 epithelial cells were treated with *Lactobacillus* strains or purified lectin protein. *L. rhamnosus* GR-1 only induced a modest 3 fold upregulation of IL13 and IL17. Of interest, IL17 has been previously suggested to control *C. albicans* vulvovaginal infections, by stimulating the production of antimicrobial peptides by vaginal epithelial cells^31^. The LGR1_Llp1 protein also moderately induced mRNA levels of the anti-inflammatory cytokines IL10 and showed a strong downregulation of IL7 mRNA. Of interest, IL-7 has been detected in the plasma of HIV-infected patients and possibly facilitates HIV-1 transmission^32^. Nevertheless, the exact cytokine signalling events in more physiological relevant conditions remain to be further substantiated.

In addition to adhesion and immunomodulating effects, we also explored the direct role of the LGR1_Llp1 lectin in pathogen exclusion, considering the fact that UPEC also shows tissue tropism for the vaginal epithelium via dedicated lectins such as FimH^14^. The lectin domains of LGR1_Llp1 were found to have a major impact on the adhesion capacity and biofilm development of the model strain *E. coli* UTI89. The adhesion capacity of this UPEC was almost reduced by half when the lectins were added in 50 μg/ml concentration. The biofilm formation was even ca. 10-fold reduced, when the lectin domain was added at the onset of the biofilm, after 1.5 h or after 24 h of biofilm formation, indicating that the lectin domain of LGR1_Llp1 can both prevent and disrupt established biofilms of this important uropathogenic pathogen, by forming loose biofilm structures. This supports previous reports of *L. rhamnosus* GR-1 inhibiting the growth and adhesion of UPEC^6^,^7^, but the exact molecular mechanism was not known till now. The localization of LGR_Llp1 within UPEC biofilms suggests that the lectin domain interact with components of the UPEC biofilm matrix. This matrix contains the polysaccharides cellulose (β-1, 4-D-glucose polymer) and colanic acid (heteropolysaccharide of glucose, galactose, fucose and glucuronic acid) in most *E. coli* strain*s*^33^,^34^. Of note, these sugars closely resemble the composition of the complex N-type glycans to which LGR1_Llp1 shows specificity. Therefore, LGR1_Llp1 might be able to bind to these exopolymeric substances and in this way destabilize the biofilm structure. This would explain the observed holes in the biofilms and the unstable biofilms formed by UPEC after adding the lectin. The clear biofilm-inhibiting effect is worth further exploration, given the prevalence of problems associated with biofilms and the increased resistance of various bacteria against antibiotics^35^. Furthermore, LGR1_Llp1 reduced the adhesion capacity of *S. aureus* Rosenbach by half, albeit not its biofilm formation. Nevertheless, taken together, our results show the inhibitory capacity of a bacterial, and more specifically a *Lactobacillus* lectin, against urogenital pathogens. It is important to note that LGR1_Llp1 did not interfere with the biofilm formation capacity of several vaginal *Lactobacillus* isolates tested, which are of crucial importance to keep the homeostasis of the vaginal environment. On the contrary, LGR1-Llp1 was even found to increase their capacity to form biofilms, suggesting that LGR1_Llp1 could play a role in maintaining a normal *Lactobacillus-*dominated vaginal microbiota or supporting their re-establishment after infections.

In conclusion, the current report describes the optimisation of genetic tools for the clinically well- documented vaginal probiotic strain *L. rhamnosus* GR-1, as well as the identification, annotation and functional analysis of *LGR1_llp1* gene as the first described adaptation factor of *L. rhamnosus* GR-1 involved in vaginal adhesion and immunomodulation. The prominent inhibitory effects of LGR1_Llp1 on biofilm formation and adhesion of UPEC and *S. aureus* holds potential for further applications related to pathogen exclusion, either alone or, in combination with other antibacterials and in the context of the probiotic *L. rhamnosus* GR-1.

## Material and Methods

### Bacterial strains, plasmids and growth conditions

The bacterial strains and plasmids used in this study are listed in Table 1. *L. rhamnosus* GR-1 wild type and the corresponding mutants were routinely grown non-shaking in de Man Rogosa Sharpe (MRS) medium (Difco)^36^ at 37 °C. *Escherichia coli* strains were grown in Luria Bertani (LB) medium (1% NaCl, 1% peptone, 0.5% yeast extract) with aeration at 37 °C^37^. *Saccharomyces cerevisiae* was grown in YPD medium (1% yeast extract, 2% peptone and 2% glucose) with aeration at 30 °C. If required, antibiotics were used at following concentrations: 10 μg/ml tetracycline, 100 μg/ml ampicillin, 5 μg/ml (for *L. rhamnosus* GR-1) or 130 μg/ml (for *E. coli*) erythromycin. The antimicrobial effect of the lectin-like protein on *E. coli* UTI89 growth was assessed by using 100-well microtiter plates (Honeycomb, Oy Growth Curves Ab Ltd.) (Bioscreen) as previously described^38^.

### DNA manipulations

Routine molecular biology techniques were performed as described before^37^. PCR primers used in this study were purchased from Integrated DNA Technologies (IDT) (Belgium) (Table 2). Enzymes for molecular biology were purchased from New England Biolabs and used according to the suppliers’ instructions. Plasmid DNA from *E. coli* was purified using QIAGEN miniprep kits. Chromosomal DNA from *L. rhamnosus* GR-1 was isolated as previously described^19^.

### Electroporation in *L. rhamnosus* GR-1

Electroporation of the replicating and integrating plasmids (Table 1) into *L. rhamnosus* GR-1 was performed as previously reported for *L. rhamnosus* GG^19^ with minor modifications. Briefly, serial dilutions (10^2^ to 10^6^ fold) were made from an overnight culture of *L. rhamnosus* GR-1 wild type into freshly prepared pre-warmed MRS medium supplemented with 2% glycine. *L. rhamnosus* GR-1 was then incubated without aeration at 37 °C. After overnight growth, ~5 ml of the selected dilution in the exponential growth phase (OD_600_ between 0.7 to 1) was added to 100 ml freshly prepared pre-warmed MRS medium supplemented with 2% glycine and incubated at 37 °C in a tightly closed 100 ml Duran bottle to minimize oxygen transfer. When the OD_600_ reached 0.2, ampicillin was added at a final concentration of 20 μg/ml to further weaken the cell wall. The culture was grown then until OD_600_ 0.4–0.5. The cells were centrifuged at 2000 × g at 4 °C and washed twice with cold electroporation washing buffer (0.5 M sucrose, 7 mM potassium phosphate-pH 7.4 and 1 ml MgCl_2_) and resuspended in 1 ml of the same buffer. Electroporation was performed in the MicroPulser electroporator (BioRad) (2 mm cuvettes) under the following conditions: 2 kV, 25 μF, 200 Ω. Subsequently, 1 ml of the regeneration buffer (fresh MRS medium supplemented with 2 mM CaCl_2_ and 2 mM MgCl_2_) was added and the cells were incubated for 3 hours at 37 °C without aeration. Finally, the cells were plated on MRS plates with appropriate antibiotics followed by incubation for 72 hours at 37 °C.

### Identification and sequence analysis of the *L. rhamnosus* GR-1 *LGR1_llp1* gene

The draft genome sequence of *L. rhamnosus* GR-1 was mined for the presence of putative lectin-like proteins by BLAST using the lectin-like protein 1 (Llp1) of *L. rhamnosus* GG. This resulted in the identification of a genomic region encoding a putative lectin-like protein of which the putative gene sequence was designated *LGR1*_*llp1*. This genomic region of *LGR1*_*llp1* and its flanking regions was submitted to Genbank under the ID accession number: KF295830.

### Construction of the *L. rhamnosus* GR-1 *LGR1_llp1* mutant CMPG10744

To determine the role of the *LGR1_llp1* gene, a corresponding knock-out mutant was constructed by double homologous recombination as previously reported for *L. rhamnosus* GG^39^. Briefly, two regions of ~1000 bp upstream and downstream of *llp1* gene, designated respectively as homologous region 1 (HR1) and homologous region 2 (HR2), were amplified by PCR. Primers were designed with restriction sites for the corresponding enzymes at the 5′ end. HR1 was amplified by PCR using primers Pro7466 and Pro7467 and subsequently cloned into pCMPG10205^39^. The pCMPG10205 plasmid is derived from pFAJ5301^40^ by ligation of a tetracycline resistance gene from *L. plantarum* MD5057 in the EcoRI site^41^. Subsequently, HR2 was amplified by PCR using primers Pro7468 and Pro7469 and cloned into the plasmid containing already HR1, resulting in plasmid pCMPG10743. This suicide vector was transferred to highly competent *L. rhamnosus* GR-1 wild type by electroporation as described above. Putative knock-out mutants resulting from double homologous recombination were selected based on resistance to tetracycline and sensitivity to erythromycin. Confirmation of DNA recombination was performed by PCR using primers Pro8018 and Pro8019 and by Southern hybridization using primers Pro7467 and Pro8018. One colony showing the correct homologous recombination event was selected for further analysis and designated as CMPG10744.

CMPG10744 was complemented by electroporation of pCMPG10746, containing the *llp1* gene, amplified by PCR with primers Pro8675 and Pro8676, ligated downstream of the *dlt* promoter in the vector pCMPG10208, resulting in the complemented strain CMPG10746.

### Construction of overexpression constructs in *E. coli* BL21 (DE3)

For heterologous expression of LGR1_Llp1 in *E. coli*, a pET a (+) system (Novagen) was used. The *LGR1_llp1* gene and the corresponding lectin domain from *L. rhamnosus* GR-1 were amplified by PCR (Table 2) and subsequently cloned into the pET-28 a (+) vector resulting in plasmids pCMPG10774 and pCMPG10775 respectively. Competent *E. coli* strain BL21 (DE3) cells were transformed with plasmid pCMPG10774 and pCMPG10775, resulting into strain CMPG10774 and CMPG10775 respectively. The recombinant *E. coli* strains for overexpression of putative lectin-like proteins were subjected to recombinant protein expression and subsequent detection by SDS-PAGE electrophoresis and Western blot.

### Production of recombinant lectins and lectin domains and their purification

The recombinant *E. coli* BL21 (DE3) strains expressing the full length lectin or the corresponding lectin domain were grown overnight in LB with 50 μg/ml Kanamycin. Each culture was diluted 100-fold in LB with Kanamycin and grown for 2 to 3 hours at 37 °C under shaking conditions until an optical density (OD) (595 nm) between 0.3 and 0.4 was reached. Then the production of recombinant protein was induced with 1 mM isopropyl β-D-thiogalactopyranoside (IPTG) and the cultures were incubated at 25 °C under shaking until an OD of 0.8 to 1 was reached. Subsequently, the cells were pelleted and suspended in non-denaturing lysis buffer (NaH_2_PO_4_ 50 mM, NaCl 300 mM, imidazole 20 mM).

The full length lectin and the corresponding lectin domain were purified from the cell lysate using affinity chromatography. The lysate was run through a HisTrapTM HP column (GE Healthcare), which contains Nickel ions embedded in a matrix of sepharose. The lectin (domain) was further purified from the eluted sample using size exclusion chromatography. The sample was applied on a HighloadTM 16/60 column packed with a matrix of SuperdexTM prep grade (GE Healthcare). Fractions containing the lectin (domain) were collected, analyzed using SDS-PAGE, pooled together and concentrated.

### SDS-PAGE and Western blot

To verify the expression of recombinant proteins, as well as the presence of pure lectin (domain) after purification steps, each fraction was separated by SDS-PAGE in Bolt 12% Bis-Tris Plus gels (Life sciences). The gel was used for a Western blot or stained with Coomassie Brilliant Blue R-250 (Bio Rad) or Sypro® Ruby protein gel stain (Invitrogen).

For performing the Western blot, the proteins from the gel were transferred to a polyvinyldifluoride (PVDF) membrane by electroblotting at 500 mA and 30 V for 1 h. For protein detection, the membrane was incubated with 0.2 μg/ml primary mouse monoclonal anti-His6 antibodies (serial no. 11922416001, Roche) in 20 ml 0.3% bovine serum albumin (BSA) (Sigma-Aldrich). Subsequently after washing, the membrane was incubated with 1:10000 diluted secondary anti-mouse antibodies conjugated with alkaline phosphatase (A3562-25ML, Sigma-Aldrich). Finally, protein detection was based on color reaction adding nitro blue tetrazolium and bromo-chloro indolyl phosphate as substrate. The reaction was stopped using 1x PBS with 25 mM ethylenediaminetetraacetic acid (EDTA).

### *S. cerevisiae* and *C. albicans* agglutination assays

To determine the lectin-binding capacity of the lectin-like domain of LGR1_Llp1, *S. cerevisiae* and *C. albicans* agglutination assays were performed as previously described^42^ with minor modifications. Briefly, overnight-grown cultures of *S*. *cerevisiae* BY4741 or *C*. *albicans* SC5314 cells were washed and suspended in PBS to final concentration of a 1% w/v cell suspension. 50 μl of these cell suspensions was added to the wells of 96-well U-bottomed plates (Cellstar® 650180, Greiner bio-one) together with 50 μl of a 400 μg/ml FITC labelled lectin domain of LGR1_Llp1, obtaining a final lectin concentration of 200 μg/ml. Strains without any lectin domain were used for negative control. The plates were incubated at room temperature for 15 minutes while gently swirling. Finally, the strains were spotted on glass slides and visualized by epifluorescence microscopy at 400-fold using the Zeiss Axio Imager Z1 microscope equipped with an AxioCam MRm Rev.3 monochrome digital camera.

### Pull-down carbohydrate binding assay using Sepharose beads

Sepharose® 6B beads (Sigma-Aldrich) were coated with 20% D-glucose, GlcNac, D-mannose, D-fucose, mannan of *S. cerevisiae* as previously described with little modification^43^,^44^. For the sugar-binding assay, 25 μl of each functionalized bead was washed with binding buffer (25 mM MES, 25 mM NaCl and 1% polyvinylalcohol) as previously described^43^. Briefly, 1 ml of binding buffer containing 100 μg/ml of the purified lectin domain was added to each bead. The mixture was then incubated at 4 °C for 2 h. The beads were washed twice with 1 ml of wash buffer and bound lectin domains were eluted by boiling the beads in SDS-PAGE loading buffer (Fermentas, Life Sciences) for 10 min at 95 °C. The bound lectin domains were resolved by SDS-PAGE through 12% polyacrylamide gels (Life Sciences), which were stained with Sypro® Ruby protein gel stain (Invitrogen) and scanned by using the Typhoon scanner (GE Healthcare Life Sciences).

### Glycan array analysis

The mammalian-glycan array version 5.2 was used to profile the sugar specificity of the lectin domain of LGR1_Llp1. The array consists of 609 glycan targets of natural and synthetic mammalian glycans with amino linkers and it is printed onto N-hydroxysuccinimide (NHS)-activated glass microscope slides (SCHOTT Nexterion), forming covalent amide linkages. The purified lectin domain of LGR1_Llp1 was labelled with FITC using FluoReporter® FITC Protein Labeling Kit (Life Technologies) according to the producer’s manual. The experiment was performed by the Consortium for Functional Glycomics (CFG, www.functionalglycomics.org).

### *In vitro* adhesion assay to a human epithelial cell lines

Adhesion assays using the VK2/E6E7 (ATCC CRL-2616™), Ect/E6E7 (ATCC CRL-2614™), End1/E6E7 (ATCC CRL-2615™), Hela (ATCC CCL-2^TM^) and Caco-2 (ATCC HTB- 37TM) cell lines were performed as previously described^45^,^46^. The adhesion ratio was calculated by comparing the number of adherent cells (CFUs) to the determined CFUs of the initially added bacterial suspension. Adhesion of *L. rhamnosus* GR-1 wild type, CMPG10744 (mutant) and CMPG10746 (complemented mutant) was tested in triplicate in three independent experiments. Alternatively, a fluorescence assay was performed as previously described^47^ with minor modifications. The FITC labelled lectin domain of LGR1_Llp1 protein was suspended in the DMEM medium in the absence of serum and antibiotics, and incubated for 1 h with the monolayer of VK2/E6E7 and Caco-2 cells grown on 13-mm coverslips. After incubation, the cells were sequentially washed three times with PBS, and fixed with 4% paraformaldehyde for 10 min. Slides were examined with a Zeiss Axio Imager Z1 microscope with an EC Plan Neofluar (X40 magnification/0.3 numerical aperture) objective (excitation 488 nm, emission 511 nm). Pictures were acquired with an AxioCam MRm and the AxioVision

In addition, the adhesion capacity of *L. rhamnosus* GR-1 wild type and CMPG10744 in the presence of the lectins concanavalin A (ConA) (Sigma-Aldrich), actinohivin (AH)^48^, griffithsin (GRFT)^49^, Nictaba^50^ and UDA^51^ was tested as previously described^46^.

### Induction of cytokine gene expression in VK2/E6E7 epithelial cells

To determine the production of different pro-inflammatory and anti-inflammatory cytokines upon co-incubation with the *L. rhamnosus* GR-1 wild type, its *LGR1_llp1* mutant and purified LGR1_Llp1 lectin domain, the vaginal VK2/E6E7 cell line was used. Cytokine expression was monitored by quantitative reverse transcription-PCR (qRT-PCR) as described previously^39^, with minor modifications. VK2/E6E7 cells growing in 12-well tissue culture plates were deprived of FCS one day prior the mRNA induction experiments. Strains were grown overnight in MRS medium and subsequently centrifuged at 2,000 × g at 4 °C for 10 min. After washing once with 1x PBS, cells were resuspended in DMEM without FCS and adjusted to a final concentration of 1 × 10^7^ CFU/ml. A 1.5-ml volume of the bacterial cell suspension was then added to the VK2/E6E7 epithelial cells for 1.5 h. Afterwards, the epithelial cells were rinsed twice with prewarmed 1x PBS. RNA was extracted from the VK2/E6E7 cells by using the high pure RNA isolation kit (Roche) following the manufacturer’s protocol. Cytokine gene expression was measured by qRT-PCR. In addition, the RT^2^ Profiler PCR Array Human Cytokines and Chemokines (Qiagen, PAHS-150Z) was used. This RT^2^ PCR array profiles the expression of 84 key secreted proteins central to the immune response.

### *In vitro* biofilm assay with selected pathogens and *Lactobacillus* species

The effect of LGR1_Llp1 on the biofilm formation capacity of *E. coli* UPI98, *S. aureus* SH1000 and *S. aureus* Rosenbach and various *Lactobacillus* strains was investigated as previously described^38^,^52^. The strains were grown on polystyrene pegs in the presence of the purified lectin domain of GR1_Llp1 at a final concentration of 50 or 200 μg/ml. The experiment was performed at least three times with eight technical repeats. The total cell count of the biofilms for *E. coli* UTI89 was also determined. *E. coli* UTI89 was allowed to form a biofilm on the bottom of polystyrene wells of 12-well culture plates (Cellstar®) and the lectin domain was added at 50 μg/ml concentration. After incubation for 48 h at 37 °C the biofilm was detached from the bottom of the wells using scrapers (Greiner bio-one) and pushed through a needle (25 G, 0.5 × 16 mm, Terumo) to dissolve cellular aggregates. The dissolved biofilms were serially diluted in PBS and plated on LB. For each strain, the experiments were performed at least three times with three technical repeats.

For the visualization of *E. coli* UTI89 biofilms, the wild-type *E. coli* UTI89 and FITC-labeled lectin domains were used. The lectin domain was added at the onset of the biofilm formation at 50 μg/ml and the biofilms were grown for 48 h. Microscopic epifluorescence imaging was performed using a Zeiss Axio Imager Z1 microscope with an EC Plan Neofluar (X40 magnification/0.3 numerical aperture) objective (excitation 488 nm, emission 511 nm). Pictures were acquired with an AxioCam MRm and the AxioVision software.

### Statistical analysis

To determine significant differences, the unequal variance t-test was applied. A P-value below 0.05 was considered as statistically significant.

## Additional Information

**How to cite this article**: Petrova, M. I. *et al.* The lectin-like protein 1 in *Lactobacillus rhamnosus* GR-1 mediates tissue-specific adherence to vaginal epithelium and inhibits urogenital pathogens. *Sci. Rep.* **6**, 37437; doi: 10.1038/srep37437 (2016).

**Publisher’s note:** Springer Nature remains neutral with regard to jurisdictional claims in published maps and institutional affiliations.

## Figures and Tables

**Table 1:** Strains and plasmids used in this study.

Strain/plasmid	Relevant genotype/description	Reference or source
*E. coli* strains		
Top10F’	F’ (*lacI*^q^, Tn^r^) *mcr*A Δ(*mrr-hsd*RMS-*mcr*BC) Φ80*Lac*ZΔM15 Δ*lac*X74 *deo*R *rec*A1 *ara*D139 Δ (*ara-leu*)7697 *gal*U *gal*K *rps*L(St^r^) *end*A1 *nup*G	Invitrogen
BL21 (DE3)	*E. coli* B F- *dcm ompT hsdS(r*_*B*_^*−*^ *m*_*B*_^*−*^) *gal λ* (DE3)	Invitrogen
*E. coli* UTI89	Wild type, clinical isolate	55
CMPG10774	*E. coli* BL21 (DE3) carrying the overexpression plasmid pCMPG10774 for secretion of the N-His6 tagged Llp1 full protein of *L. rhamnosus* GR-1, Km^R^	This study
CMPG10775	*E. coli* BL21 (DE3) carrying the overexpression plasmid pCMPG10775 for secretion of the N-His6 tagged Llp1 lectin-like domain from *L. rhamnosus* GR-1, Km^R^	This study
*L. rhamnosus* GR-1 strains		
Wild type	Human urethra isolate	ATCC 5582
CMPG10744	*llp1* knock-out mutant of *L. rhamnosus* GR-1; *llp1::tet*^*R*^	This study
CMPG10746	CMPG10744 mutant complemented by electroporation of pCMPG10746	This study
*Lactobacillus* strains		
*L. reuteri* RC-14 ATCC 55845	Wild-type, female urethra isolate	56
*L. crispatus* NCIMB 4505	Wild-type, human vaginal isolate	57
*L. jensenii* ATCC 25258	Wild-type, human vaginal isolate	58
*L. gasseri* ATCC 33323	Wild-type, human vaginal isolate	58
*L. plantarum* CMPG5300	Wild-type, human vaginal isolate	59
Other strains		
*S. cerevisiae* BY 4741	MATa; his3Δ 1; leu2Δ 0; met15Δ 0; ura3Δ 0	60
*C. albicans* SC5314 (ATCC MYA-2876)	Wild type, human clinical isolate	61
*S. aureus* SH1000	*rsbU* positive derivative of *S. aureus* 8325-4	62
*S. aureus* Rosenbach (ATCC 33591)	Wild type, clinical isolate	ATCC
Plasmids		
pLAB1301	*E. coli* – *Lactobacillus* shuttle vector, Ery^R^, Amp^R^	18
pEM40	pUC19E-derived integration vector (*attB* located at the 3′ end of the tRNA^Leu^ locus) containing a 1.6-kb int-*attP* cassette of phage A2; Ery^R^, Amp^R^	17
pET 28 a(+)	KmR, T7 lac, N and C-terminal His Tag	Novagen
pCMPG10205	Cloning vector; pUC18 containing tetracycline resistant cassette from pGK13 in the BspHI site	39
pCMPG10208	pLAB1301 derivative driven by promoter of the *dlt* operon of *L. rhamnosus* GG, Amp^R^, Ery^R^	63
pCMPG10743	pCMPG10205 derivative used to inactivate the *llp1* gene by insertion of a *tetR* marker via double homologous recombination (for details, see text)	This study
pCMPG10746	pCMPG10208 derivative containing the *llp1* gene (2040 bp) in the *XmaI/SacI* site, Amp^R^, Ery^R^	This study
pCMPG10774	pET28 (a+) derivative carrying the N-His6 tagged *llp1* gene of *L. rhamnosus* GR-1 in the *Sal*I/*Not*I site, Km^R^	This study
pCMPG10775	pET28 (a+) derivative carrying the N-His6 tagged lectin-like domain with 22 amino acids extension of the *llp1* gene of *L. rhamnosus* GR-1 in the *Sal*I/*Not*I site, Km^R^	This study

Tet^R^, tetracycline resistance; Em^R^, erythromycin resistance; Ap^R^, ampicillin resistance, Km^R^ kanamycin resistance.

**Table 2:** List of primers used in the study.

Primer	Primer sequence (5′-3′)	Restriction site	Remarks
Pro564	AGCAGGACGAGAAAGCAATGAATGT	/	Forward primer to check pEM40 integration in *Lactobacillus* genome
Pro565	GCCGGTGTGGCGGAATTGGCAG	/	Reverse primer to check pEM40 integration
Pro7129	ATGTTCATGTAATCACTCCTTCTTAATTAC	/	Reverse primer to check insertion in multiple cloning site pLAB1301
Pro7130	ATAGGCTCCAAAAGGAGCCTTTAATTGTA	/	Forward primer to check insertion in multiple cloning site of pLAB1301
Pro7466	ATCCCGGGCCAAAATCATCCGTG	*XmaI*	Forward primer HR1 *llp1* gene LGR-1
Pro7467	ATCCCGGGCATGATCGTCACTCCT	*XmaI*	Reverse primer HR1 *llp1* gene LGR-1. Used for Southern hybridisation
Pro7468	ATGCGGCCGCGTGAGTCGAGTAAGCA	*NotI*	Forward primer HR2 *llp1* gene LGR-1
Pro7469	ATGTCGACTGCTAGCGGTATATTCA	*SalI*	Reverse primer HR2 *llp1* gene LGR-1
Pro8018	TTACACTCCGACTTCTAACCGC	/	Forward primer to check *llp1* replacement with Tc^R^ cassette. Used for Southern hybridisation
Pro8019	CTAATCAGCGATGCTTAGTCG	/	Reverse primer to check *llp1* replacement with Tc^R^ cassette
Pro8675	ATCCCGGGATGAAGAAGTGCGGCTA	*XmaI*	Forward primer complementation *llp1*gene
Pro8676	ATGAGCTCTCACTGAGGAGCGTT	*SacI*	Reverse primer complementation *llp1* gene
Pro5880	CACCGTCGACCGAAGAAGAAATATTCA	*SalI*	Forward primer for full length llp1 gene for pET28 a(+)
Pro5881	ACTGGCGGCCGCTTAAGGCATAGGAGTAG	*NotI*	Reverse primer for full length llp1 gene for pET28 a(+)
S&P-00517	ATGTCGACAAGGGTGGCCGTCATCGTCAGG	*SalI*	Forward primer upstream of lectin-like domain of *llp1* gene
S&P-00518	ATGCGGCCGCTTAATCTTCTACCTTCAAATGCGTG	*NotI*	Reverse primer upstream of lectin-like domain of *llp1* gene
S&P-0044	TGGCAGCAGCCAACTCAGCTT	*/*	Reverse primer for MCS of pET28 a(+)
S&P-0045	TATAGGCGCCAGCAACCGCA	*/*	Forward primer for MCS of pET28 a(+)
